# Beyond psychotropics: critical strategies for supporting youth in foster care

**DOI:** 10.1186/s13034-025-00874-9

**Published:** 2025-02-28

**Authors:** Nicole L. Hadler, Gerrit I. van Schalkwyk

**Affiliations:** 1 Department of Mental Health, Naval Medical Center San Diego, 34800 Bob Wilson Drive, San Diego, CA 92116 USA; 2 Yale University Child Study Center, New Haven, CT USA

## Background

Around half of children living in foster care worldwide have been diagnosed with a mental health disorder [1, 2]. Children in foster care are diagnosed with attention deficit hyperactivity disorder (ADHD), depression, anxiety, and disruptive mood and impulse control disorders at rates three to four time higher than non-foster children from similar socioeconomic and educational backgrounds [2]. They are four times more likely to attempt suicide [2]. A product of the high burden of mental illness is a higher rate of psychotropic medication prescription to include antipsychotic medications, even when controlling for diagnostic and demographic variables [2, 3]. 

The high rate of psychotropic prescribing is a consequence of the clinical complexity of this population. Although it is broadly recognized that psychosocial interventions should be considered first in most clinical contexts, there are significant barriers to their initiation and effective delivery. The prominence of disruptive behaviors may limit ability for consistent engagement in therapy, and create greater urgency around the need for immediate action such as in the form of a medication. The unique and pervasive nature of attachment issues in this population requires specific considerations in the provision of care that recognize the potential limitations and vulnerabilities of the child-caregiver relationship in these individuals. Complex trauma exposure may complicate the presentation of each other psychiatric difficulty and require more informed responses from family and clinical teams. And the significantly elevated number of caregiver transitions may have broad developmental impacts and present recurrent sources of interpersonal stress and loss. This article provides an overview of four clinical features that can be particularly burdensome for foster children and interfere with delivery of effective psychiatric care, as well as guidance on how the child psychiatrist can most appropriately respond to the foster youth’s needs in each scenario.

## Disruptive behaviors

*Foster parents of a 6 year old boy arrive late to an appointment*,* appear exhausted*,* and say they are at their wits’ end with the child’s behavior. “We couldn’t get him out the car today. He just doesn’t listen no matter what we do”.*

Foster children are at disproportionate risk of developing behavioral problems such as defiance or aggression; furthermore, behavioral problems can be a source of substantial frustration for families and are a known risk factor for placement disruptions [4, 5]. Misunderstood disruptive behaviors can leave foster children in a vicious cycle of further placement instability and worsening of behaviors and mental health outcomes. The clinical interview of a child presenting with behavioral problems should entail a comprehensive social history including trauma exposure and attachment disruptions. Maintaining an open, patient-centered approach throughout the clinical encounter can promote the child’s sense of safety, and it can also serve as a model for future interactions between the child and caregiver.

A psychiatrist can work with the child to identify potential triggers leading up to the disruptive behavior, to include trauma-related triggers. Guiding the child to label their experienced emotions can help the child process their emotional state, develop vocabulary and confidence to express their feelings, and improve their sense of security and self-worth when they experience different emotions. It is important for the psychiatrist to include the caregiver in discussions about emotion identification and expression, to promote a culture of emotional safety not exclusive to the medical setting but also in the home environment.

While the child should remain at the focus of treatment, caregiver involvement is crucial in navigating behavioral problems. Clinicians should reframe the disruptive behaviors using a trauma informed lens, highlighting how exposure to adverse childhood experiences can impair a child’s emotional regulation and stress response. Furthermore, reminders of trauma and past experiences of grief and loss can lead to avoidance or acting out behaviors. Recognizing that the source of these behaviors is not from a place of malice toward the current family, but rather from past trauma and loss, can help parents build empathy when responding to their child’s behaviors.

Providing parents with behavior management tools that can be practiced in the home can empower families to start to address these behaviors, especially if there are barriers for the child engaging in regular therapy. The psychiatrist can collaborate with the patient and parents on behavior management strategies, emphasizing the importance of avoiding harsh disciplinary measures and praising for positive behaviors. Basic cognitive behavioral therapy (CBT) skills can be highlighted in the office, including relaxation techniques such as deep breathing, problem solving strategies to explore the behavior trigger and potential solutions with the child, and replacing maladaptive behaviors with assertive communication and other more socially appropriate behaviors. If additional resources are needed, Multidimensional Treatment Foster Care programs have been established to provide more intensive and proactive support for the youth and foster parents, such as providing caregiver training focused on positive reinforcement and prosocial behaviors [6]. 

## Unstable attachments

*Ian is described by his foster parents as ‘overly touchy’ and ‘impulsive’. They note he will hug strangers in public places*,* and doesn’t understand ‘personal space’.*

Attachment theory defines how much is required from a healthy parent-child dyad to support the development of a child who comes to feel secure in their connection to their family, with the necessary foundation for exploring the world with security. A good outcome requires concerted efforts even in the best of circumstances, when there is consistency among primary caregivers and few disruptions to the who and where of a child’s experience. The task is greatly complicated in the case of foster youth, for whom the lack of a stable and consistent caregiving presence may predispose to disruptions in social, cognitive, and emotional development to include subsequent patterns of attachment [7]. It is important to highlight to families that risk factors for an attachment disorder include loss of a caregiver, multiple foster home placements, and exposure to neglect or abuse, which places children in foster care at an especially heightened risk for these disorders [7]. 

A child’s behaviors and interactions with their caregiver, influenced by previous caregiver-child relationships, can be particularly frustrating for the caregiver who is unable to connect with the child on a level they had hoped. The frustration may be amplified when parents view themselves as dedicated, consistent, and present, but see results that reflect a mixture of their own efforts, those of previous caregivers, and the inherent disadvantages of multiple caregiver transitions. Not only should a psychiatrist provide psychoeducation on the origins of attachment disorders to provide context to the family’s experience, but the psychiatrist can also emphasize the high potential for the formation of a secure attachment. Even after experiences of serious neglect, children can go on to develop stable attachments if their subsequent caregiver has the patience and appropriate tools in place [8]. 

Psychiatrists can educate parents on the basic tools for secure attachment formation which should be executed especially purposefully for a foster child. The cornerstone is for the parent to act as a reliable, consistent, emotionally available caregiver. Responding promptly and consistently to a child’s distress builds trust and the child’s sense of safety. Psychiatrists should identify potential barriers to a caregiver’s responsiveness to their child’s needs, such as an untreated mental illness in the caregiver themselves, or the caregiver’s own history of insecure attachments. Psychiatrists should stress the importance of a predictable routine, generous validation and praise, and age-appropriate autonomy. Setting realistic expectations on the gradual rather than immediate formation of a stable attachment can encourage patience and perseverance during setbacks.

## Complex trauma exposure

*A 12 year old girl in foster care has not bathed in several weeks. Her foster parents report that she ‘shuts down’ when they insist that she take a shower.*

Traumatic experiences are the rule rather the exception among foster children, and they are associated with PTSD, anxiety disorders, substance use, disruptive behaviors, and many other conditions including mood disorders and attachment disorders [9]. Furthermore, a child with PTSD can often be misdiagnosed as having other psychiatric or neurodevelopmental disorders such as ADHD, conduct disorder, and autism spectrum disorder [10]. Consideration for features and sequelae of developmental trauma disorder, a proposed diagnosis stemming from exposure to traumatic victimization and/or caregiver disruptions, may assist in a psychiatrist’s conceptualization of children whose symptomatology is difficult to capture within the existing trauma-related classification systems [11]. Psychiatrists should discuss the high prevalence of trauma exposures among foster youth and the varying types of trauma that can occur within a home to include neglect, abuse, and domestic violence. Foster children are at risk of exposure to repeated or prolonged traumatic experiences, which can lead to more severe and complex effects on the child’s psychosocial and neurobiological development. Psychiatrists should screen every foster child for trauma; the National Child Traumatic Stress Network (NCTSN) created the Child Welfare Trauma Referral Tool specifically for foster children [12]. The NCTSN also provides resources for both providers and families on trauma informed care. Furthermore, the Child Welfare System Systems Based Practice Guidelines highlights five core principles of trauma informed care: safety, trustworthiness, choice, collaboration, and empowerment [13]. 

The mainstay intervention for trauma related disorders is trauma focused therapy, with trauma-focused CBT being one of the most widely studied and implemented for children [14, 15]. Aside from encouraging early and frequent engagement in trauma therapy, a psychiatrist can provide additional in-office guidance. Psychiatrists should ensure the home provides an emotionally safe environment for the child by encouraging the child’s access to a safe physical space such as their bedroom which is respected as the child’s personal space, provision of age-appropriate choices and autonomy, recognition of and praise for the child’s strengths, and modeling of emotion expression and regulation by caregivers. During early stages of trauma treatment, it may be beneficial for the psychiatrist to have more frequent follow-up visits with the child to provide a consistent support. Ensuring there is space for the child to talk about trauma, even if not the focus of the visit, can empower the child and family to communicate any ongoing concerns.

## Caregiver transitions

*A 9-year-old foster youth is being managed with medications for aggressive outbursts related to autism spectrum disorder. At a follow-up appointment*,* his caregiver informs the psychiatrist that she has submitted a 14-day notice to have the child removed from her home*,* stating she can no longer handle the child’s behavioral needs.*

Over half of children in foster care for at least two years have experienced three or more out-of-home placements [4]. Each new placement disrupts prefrontal cortex development leading to deficits in executive functioning, which can be seen in disruptive behavior disorders and ADHD [4]. A psychiatrist should understand the risk factors for placement disruption which include behavioral problems, longer duration in foster care, multiple prior placements, adolescent age, and negative foster parent experiences [16, 17]. It is vital that the psychiatrist be proactive in addressing the needs of both the caregiver and child to maximize the likelihood of placement stability. Collaboration with the child’s caseworker, prompt referrals to therapy focused on the caregiver-child relationship such as parent-child interaction therapy, and provision of resources to bolster caregiver training and well-being such as Keeping Foster and Kin Parents Supported and Trained, are all potential tools a child psychiatrist can provide to support placement stability [18]. A set of clear and practical guidance has yet been established as a tool for a psychiatrist to directly mitigate the risks of caregiver transitions, which are deeply detrimental for the child.

When a caregiver transition is imminent or in progress, a provider can encourage the caregiver to have an age-appropriate discussion with the child about the upcoming transition, with emphasis on avoiding blaming the child for the placement disruption. The caregiver can display a calendar as a visual aid to keep the child informed and aware of the timeline. The transition should be as gradual as possible, with pre-placement visitations encouraged. A provider should encourage the remaining parts of the child’s support network and routine, such as school, therapist, mentors, and biological family visits, to remain as constant as possible. The previous family can also be encouraged to remain in contact with the child to help preserve the child’s sense of self-worth and to allow the child to continue to process this transition, if the former family is able to serve as predictable, safe adults during this transition.

## Conclusion

The high burden of mental health symptoms among foster children is nearly inevitable given the extent of trauma and loss experienced by this population. The high burden of psychotropic use, however, can be ameliorated by a more comprehensive and proactive approach to their psychosocial needs. Numerous states within the United States have released initiatives to mitigate psychotropic prescribing; [19, 20] there is not yet unified national or international guidance for psychiatric providers when working with foster children, focused on addressing behavioral problems, attachment issues, trauma exposure, and caregiver transitions. A child psychiatrist’s attunement to the impact of caregiver transitions on long-term psychiatric and psychosocial outcomes is of particular importance, and targeted support toward the child and caregivers could be a great contribution to placement stability. It is imperative for providers to be familiar with the basic tools to guide foster children and families through these challenges, and to lean on a multidisciplinary team of therapists, social workers, and case managers to help foster families navigate the widespread supports and interventions available for their children.

## Data Availability

Data sharing is not applicable to this article as no datasets were generated or analyzed in this manuscript.
